# Long-term efficacy of total versus posterior partial fundoplication in patients with gastro-oesophageal reflux disease: a systematic review and meta-analysis

**DOI:** 10.1308/rcsann.2023.0046

**Published:** 2023-10-16

**Authors:** DV Peristeri, H Room, D Tsironis, G Vasilikostas, A Wan

**Affiliations:** St George’s University Hospitals NHS Foundation Trust, UK

**Keywords:** Long-term outcomes, Total fundoplication, Posterior partial fundoplication, Nissen, Toupet, Gastro-oesophageal reflux disease, Antireflux surgery

## Abstract

**Introduction:**

Laparoscopic fundoplication remains the standard treatment for patients with severe gastro-oesophageal reflux disease (GORD). Multiple randomised controlled trials (RCTs) have compared the two most commonly performed surgical techniques, total and posterior partial fundoplication (Nissen [NF] and Toupet [TF]), in terms of symptom control and treatment failure in patients without subsequent dysmotility disorders. We aimed to conduct a systematic review and meta-analysis of these two techniques with regard to the long-term effect on reflux control and associated dysphagia.

**Methods:**

The MEDLINE^®^, Embase^®^, PubMed^®^ and Cochrane Library databases were searched, and all the relevant published RCTs were shortlisted according to the inclusion criteria. The summated outcomes of long-term results relating to the recurrence of GORD and dysphagia were evaluated in a meta-analysis using RevMan software.

**Results:**

Eight studies (all RCTs) on 1,545 patients undergoing NF or TF were eligible for inclusion in this meta-analysis. There were 799 patients in the NF group and 746 in the TF group. In the random effects model analysis, the incidence of long-term recurrence of GORD was not statistically different between the NF and TF cohorts (odds ratio [OR]: 0.69, 95% confidence interval [CI]: 0.34–1.41, z=1.01, *p*=0.31). However, the incidence of long-term dysphagia was statistically lower in the TF group (OR: 2.92, 95% CI: 1.49–5.72, z=3.13, *p*=0.002) with low between-study heterogeneity (I^2^=0%).

**Conclusions:**

The findings of this systematic review and meta-analysis on symptomatic GORD appear to be in favour of partial posterior fundoplication (TF) as the optimal treatment. It provides equivalent outcomes in reflux symptom control with a lower risk of postoperative dysphagia compared with total fundoplication (NF).

## Introduction

Gastroesophageal reflux disease (GORD) is one of the most common gastrointestinal diseases, defined according to the Montreal consensus as “a condition which develops when the reflux of stomach contents causes troublesome symptoms and/or complications”.^1^ The prevalence of GORD is increasing, particularly in developed countries, where up to 20% of the population seems to be affected.^2,3^ Surgical intervention is generally indicated in patients with moderate to severe GORD, either where symptoms are insufficiently controlled by medical therapy or owing to patient choice.^4,5^ Surgery, most commonly a laparoscopic fundoplication, has a long-term failure rate of 10–15%.^6,7^ Operative failure is usually defined as recurrent reflux symptoms and/or dysphagia, which could have a negative impact on the patient’s quality of life, and a few cases may require revisional surgery.^8,9^

Standard surgical fundoplication techniques in GORD patients include total fundoplication (Nissen [NF]), posterior partial fundoplication (Toupet [TF]) and anterior partial fundoplication (Dor/Watson). These procedures vary in efficacy and durability as well as in adverse side effect profiles. At one end of the spectrum, NF (where a total 360° wrap surrounds the oesophagus) has been noted to be highly effective in relieving GORD symptoms and is the most durable among the procedures. Nevertheless, evidence shows that NF is also associated with the most significant potential for adverse effects, such as dysphagia, difficulty in vomiting and gas bloating.^10^ TF (a posterior partial 270° wrap) was introduced to counteract these side effects.^11^ A partial 180° anterior wrap (Dor/Watson) is generally used for patients with associated motor abnormalities.^12^

An ongoing discussion has focused on the ideal approach for patients without preoperative oesophageal motility disorders, including durable reflux control, as well as minimal postoperative dysphagia and gas-related symptoms. The comparison between NF and TF is not new but still remains a complex topic.^7,13^

Many trials and meta-analyses have demonstrated that a well constructed TF could result in reflux control similar to that of a well performed NF, with fewer adverse effects.^7,14,15^ However, these trials were of limited quality and power. Moreover, most meta-analyses were based on studies with a short-term follow-up of fewer than 12 months after surgery. Recently, several RCTs with larger sample sizes and extended follow-up data have been published comparing the long-term efficacy and adverse events of both total and posterior partial fundoplication.^16–23^ The aim of the present study was to systematically review and analyse recent RCTs comparing NF and TF with regard to the long-term effect of more than two years of GORD symptoms and dysphagia in patients with normal oesophageal motility so as to determine which procedure should be regarded as the surgical therapy of choice.

## Methods

This review was undertaken in accordance with the PRISMA (Preferred Reporting Items for Systematic reviews and Meta-Analyses) guidelines.^24^ An electronic search of the MEDLINE^®^, Embase^®^, PubMed^®^ and Cochrane Library databases was conducted for relevant articles published between August 2000 and August 2022. Medical Subject Headings terms pertinent to the target objective were employed in the search to find relevant studies. Boolean operators (AND, OR, NOT) were used to optimise the search results. Data of interest were abstracted by the first author and cross-checked by the senior author for potential inclusion in the review. The references from the included papers were also searched to identify additional trials. Grey literature was omitted.

### Study selection

Only RCTs directly comparing NF with TF in adult patients (>18 years) without any preoperative background of oesophageal dysmotility were included in the review. Studies with a minimum follow-up duration of 24 months were considered eligible. Those comparing medical treatments versus surgery were excluded, as were quasiexperimental studies, cohort studies and case controlled studies. There were no further restrictions by study site or country. Studies were considered eligible irrespective of language or hospital of origin. Two independent reviewers screened studies for eligibility. The primary outcome of interest was the recurrence of GORD and the secondary outcome was long-term postoperative dysphagia.

### Risk of bias

The Cochrane Collaboration’s tool for assessment of risk of bias among studies was employed.^25^ The following criteria were assessed: random sequence generation, allocation concealment, blinding of participants and personnel, blinding of outcome assessment, incomplete outcome data, selective reporting and other sources of bias. The risk of bias assessment was summarised using RevMan 5.4 (Nordic Cochrane Centre, Copenhagen, Denmark).^26^

### Evidence synthesis

RevMan 5.4 was also employed for statistical analysis.^26,27^ The odds ratio (OR) with a 95% confidence interval (CI) was used to express the summated outcomes for binary data and the standardised mean difference with a 95% CI was presented for continuous data. Long-term effects on dysphagia and recurrence of GORD were compared between the two arms using ORs with a 95% CI. The random effects model was employed to calculate the combined outcomes of dichotomous variables.^28,29^ Heterogeneity among the included studies was explored with the chi-squared test (significance set at *p*<0.05) and quantified with the I^2^ statistic.^30^ An I^2^ value of ≤30% indicated low heterogeneity.^31^ If the standard deviation was not available, it was calculated according to the guidelines of the Cochrane Collaboration.^27^ The Mantel–Haenszel method was used to calculate ORs in the random effects model analysis.^32^ Only RCTs that were clinically homogenous and that directly compared the same two surgical procedures were pooled.^33^

In a sensitivity analysis, 0.5 was added to each cell frequency for trials in which no event occurred in either the NF or TF group, as per Deeks *et al*.^33^ If the standard deviation was unavailable, it was calculated according to the guidelines provided by the Cochrane Collaboration.^27,28^ This process involved assumptions that both groups had the same variance, which may not have been confirmed, and variance was estimated either from the range or from the *p*-value. The estimate of the difference between both surgical techniques was pooled, depending on the effect weights in results determined by each trial estimate variance. A forest plot was generated for graphical display of the results. The square around the estimate stood for the accuracy of the estimation (sample size) and the horizontal line represented the 95% CI. The methodological quality of the included RCTs was assessed as per Jadad *et al* and Chalmers *et al*.^34,35^

## Results

**Figure 1:**
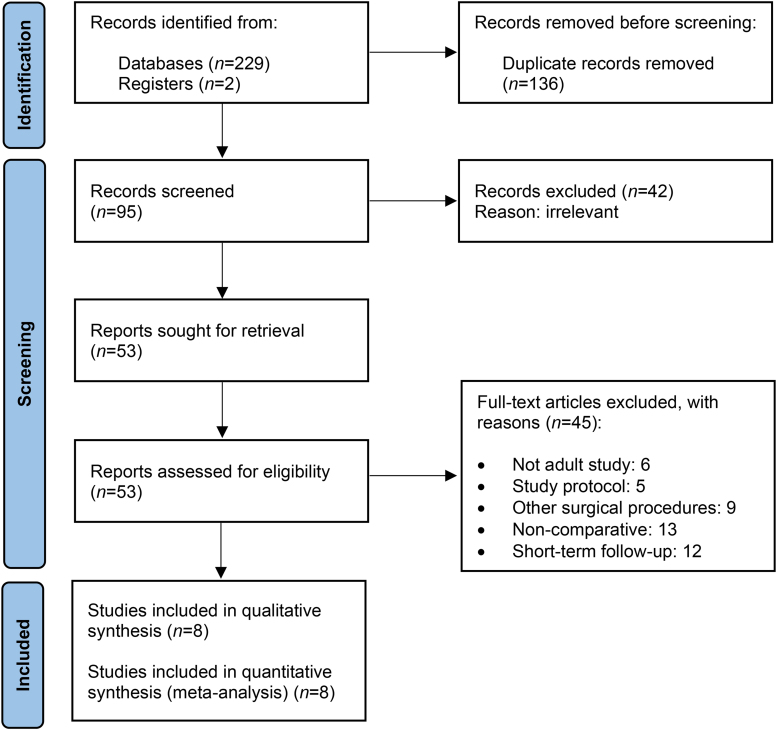
Study selection

The initial literature search identified 231 records. Following removal of duplicate records and screening of full-text articles for eligibility, 45 articles were excluded for not meeting the inclusion criteria. Consequently, eight studies, all RCTs that met the aforementioned criteria, were included in this review (Figure 1).^16–23^

### Study characteristics

The characteristics and treatment protocols of the included RCTs are summarised in Tables 1 and 2. A total of 1,545 patients were reported, of which 799 were allocated to the NF group and 746 to the TF group. All papers directly compared long-term outcomes (≥24 months) of NF versus TF. The duration of follow-up ranged between 2 and 12 years. Six trials calculated patients’ body mass index, which was similar and ranged from 26kg/m^2^ to 34kg/m^2^.^16–19,21,23^

**Table 1:** Characteristics of the included studies

Study	Study design	Country	Number of participants	Mean age in years (NF vs TF)	Mean body mass index in kg/m^2^ (NF vs TF)	Intervention groups
Analatos, 2022^16^	RCT	Sweden	310 (151 NF, 159 TF)	66±11.2	27.8±3.8 vs 27.4±3.9	NF vs TF
Guérin, 2007^17^	RCT	Belgium	140 (77 NF, 63 TF)	Not available	27 vs 26.6	NF vs TF
Gunter, 2017^18^	RCT	Wisconsin, US	316 (161 NF, 155 TF)	51.6±13.8 vs 56.6±14.6	29.98±4.97 vs 30.02±5.43	NF vs TF
Mickevičius, 2013^19^	RCT	Lithuania	124 (60 NF, 64 TF)	49.2±14.4 vs 54.8±12.6	27.7±4.6 vs 29.3±3.2	NF vs TF
Qin, 2013^20^	RCT	China	383 (215 NF, 168 TF)	56.3	Not available	NF vs TF
Shaw, 2010^21^	RCT	South Africa	100 (50 NF, 50 TF)	45.2 (28–72) vs 45.6 (25–67)	29.3±5.2 vs 29.2±5.2	NF vs TF
Strate, 2008^22^	RCT	Germany	100 (50 NF, 50 TF)	56.3	Not available	NF vs TF
Wang, 2015^23^	RCT	China	80 (40 NF, 40 TF)	57±13.2 vs 57±10.8	23.5±2.7 vs 2.5±3.4	NF vs TF
NF = Nissen fundoplication; RCT = randomised controlled trial; TF = Toupet fundoplication						

**Table 2:** Treatment protocols adopted in the included studies

Study	Primary outcome	Secondary outcome	Dysphagia rates	Recurrence of GORD	Follow-up duration
Analatos, 2022^16^	QoL, recurrence of GORD and PPI consumption	Dysphagia scores for solid and liquid food items after >15 years	NF: 2/149 (1.3%) TF: 1/158 (0.6%)	NF: 42/149 (28.2%) TF: 38/158 (24.1%)	15 years
Guérin, 2007^17^	Recurrence of GORD	Invaliding symptoms including dysphagia (solids and liquids)	NF: 2/77 (2.6%) TF: 0/63 (0%)	NF: 4/77 (5.2%) TF: 3/63 (4.8%)	3 years
Gunter, 2017^18^	Recurrence of GORD	QoL outcomes including dysphagia	NF: 5/161 (3.1%) TF: 3/155 (1.9%)	NF: 61/161 (37.9%) TF: 108/155 (69.7%)	5 years
Mickevičius, 2013^19^	Intensity of heartburn, dysphagia, gas bloating and presence of oesophagitis	Recurrence of GORD or persistent dysphagia	NF: 6/60 (10.0%) TF: 2/64 (3.1%)	NF: 7/60 (11.7%) TF: 8/64 (12.5%)	5 years
Qin, 2013^20^	Incidence of postoperative dysphagia and abdominal distension	Recurrence of GORD	NF: 3/215 (2.4%) TF: 0/168 (0%)	NF: 0/215 (0%) TF: 18/168 (10.7%)	5.6 years
Shaw, 2010^21^	Recurrence of GORD	Dysphagia and dysmotility	NF: 2/47 (4.3%) TF: 0/48 (0%)	NF: 8/47 (17.0%) TF: 8/48 (16.7%)	5 years
Strate, 2008^22^	Recurrence of GORD and satisfaction	Dysphagia	NF: 6/50 (12.0%) TF: 3/50 (6.0%)	NF: 13/50 (26.0%) TF: 8/50 (16.0%)	2 years
Wang, 2015^23^	Improvements in symptom scores and QoL	Dysphagia and recurrence of GORD	NF: 9/40 (22.5%) TF: 2/40 (5.0%)	NF: 7/40 (17.5%) TF: 11/40 (27.5%)	3 years
GORD = gastro-oesophageal reflux disease; PPI = proton pump inhibitor; QoL = quality of life; NF = Nissen fundoplication; TF = Toupet fundoplication					

All included studies identified the two types of surgery performed: total (NF) versus posterior partial (TF) fundoplication. These trials were published between 2007 and 2022, and publications originated from Europe,^16,17,19,22^ China,^20,23^ the US^18^ and South Africa.^21^ GORD was the first inclusion criterion across studies. No RCTs considered patients with large hernias (>5cm). Only one trial included patients with oesophageal dysmotility and compared these with patients with normal oesophageal motility.^21^ All other RCTs excluded patients with dysmotility disorders.

### Recurrence of GORD symptoms

**Figure 2:**
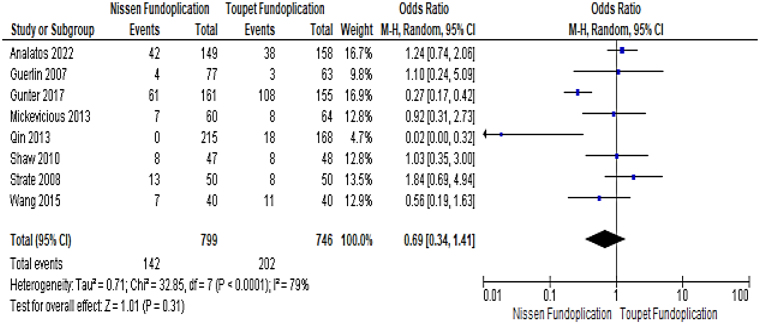
Forest plot comparing results for recurrence of gastro-oesophageal reflux disease

The postoperative reappearance of GORD symptoms generally includes heartburn, regurgitation, chest pain, endoscopic report of oesophagitis or recommencement of proton pump inhibitors. Heartburn is the most typical symptom and reappearance of heartburn was considered to indicate recurrence in all included studies. Reappearance of GORD symptoms was reported in 17.77% of patients following NF (142/799) and 27.07% (202/746) following TF (OR: 0.69, 95% CI: 0.34–1.41, *p*=0.31) (Figure 2). Excessive heterogeneity existed so the random effects model was used to pool data.

### Postoperative dysphagia

**Figure 3:**
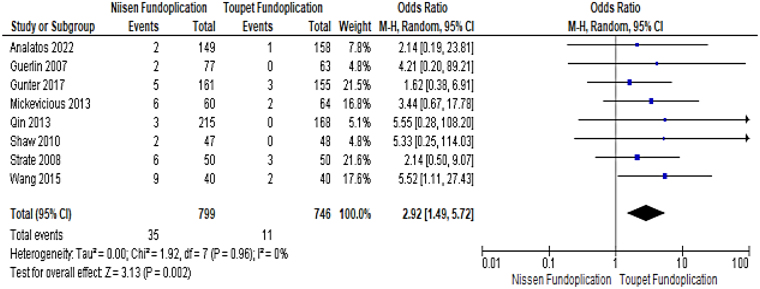
Forest plot comparing results for postoperative dysphagia

All RCTs also reported on the long-term outcome of postoperative dysphagia in both groups. Long-term dysphagia was defined as persisting for more than 24 months following laparoscopic NF or TF. NF was associated with a significantly higher prevalence of postoperative dysphagia (4.38% [35/799] vs 1.47% [11/746], OR: 2.92, 95% CI: 1.49–5.72, *p*=0.002) (Figure 3).

## Discussion

Despite numerous trials comparing different types of fundoplication, the optimal surgical treatment for GORD remains under debate. In this analysis, NF and TF were found to have equivalent long-term GORD symptom control although TF was associated with fewer cases of long-term postoperative dysphagia.^20^

The 2021 guidelines from the Society of American Gastrointestinal and Endoscopic Surgeons suggest choosing between a complete or posterior partial fundoplication based on the patient’s values and preferences.^36^ Patients without known oesophageal dysmotility and who value the resolution of reflux symptoms over the risk of dysphagia should undergo a complete rather than a partial fundoplication. However, recent meta-analyses could not agree on which procedure is most suitable, offering fewer complications and long-lasting effects in GORD patients.^14,15,37–39^

Tian *et al* concluded that TF might be a better surgical approach for GORD, with a lower rate of adverse postoperative outcomes and equal effectiveness with NF.^14^ Nevertheless, they only included trials with a short-term follow-up of under 12 months. Moreover, the trials did not directly compare NF versus TF. Another well conducted meta-analysis by Tan *et al* found that both operations were safe and effective, with similar results in terms of postoperative dysphagia and recurrence rates.^37^ However, the included studies did not compare similar outcomes. Their results, likewise, could not be considered long-lasting as the trials had a short follow-up period of fewer than 12 months. Du *et al* also concluded that NF and TF have similar results with regard to GORD symptom control.^38^ The follow-up duration was particularly short in most of their included studies, making their results less reliable on a long-term basis.

One of the largest meta-analyses, conducted by Andreou *et al* and including 29 RCTs, concluded that TF has a better outcome than NF, especially relating to postoperative dysphagia.^39^ The authors mentioned that NF was also associated with equivalent GORD symptom control when compared with all partial fundoplications. It is worth highlighting that their meta-analysis was not specifically related to TF versus NF; all comparisons made also included multiple surgical and medical treatments. Equally important is the fact that the duration of follow-up was less than five years in most trials, which makes conclusions about the long-term efficacy of these procedures less applicable. Some of the included papers reported a high risk of bias, which further weakens the power of their results. In addition, some studies included patients with preoperative oesophageal motility disorders.

Finally, a meta-analysis from 2010 by Broeders *et al* concluded that TF has superior results to NF both in terms of reflux control and dysphagia.^15^ Nevertheless, the methodological quality of the included studies was low and most had only a short follow-up period (<18 months).

Our analysis included eight RCTs, the majority of which appeared to provide high-quality results (Table 3). To our knowledge, our study is the first to focus purely on recent high-quality RCTs directly comparing NF versus TF in GORD patients without known oesophageal dysmotility disorders and with a follow-up duration of more than 24 months. As surgical techniques and patient populations are continuously evolving, this provides a contemporaneous and high-quality synthesis of the latest evidence. The most recent RCTs represent the optimal study design for pooled results with higher statistical power.

**Table 3:** Quality of the included studies

Study	Randomisation technique	Power calculations	Blinding	Intention-to-treat analysis	Concealment
Analatos, 2022^16^	Computer generated	Yes	Yes	Yes	Yes
Guérin, 2007^17^	Not applicable	Not available	Yes	Yes	Yes
Gunter, 2017^18^	Not applicable	Not available	Yes	Not available	Yes
Mickevičius, 2013^19^	Sealed envelopes	Yes	Yes	Yes	Yes
Qin, 2013^20^	According to the parity of the patient’s hospital number	Not available	Yes	Yes	No
Shaw, 2010^21^	Computer generated	Yes	Yes	Yes	Yes
Strate, 2008^22^	Not applicable	Not available	Yes	Yes	Yes
Wang, 2015^23^	Computer generated	Yes	Yes	Yes	Yes

### Study limitations

Inevitably, this study has some limitations. First, owing to the differences in follow-up duration between the included RCTs, there was a degree of heterogeneity observed, which might undermine the quality and legitimacy of the obtained results. Second, the diagnosis of recurrent GORD symptoms is not standardised in clinical practice or in the literature. The most robust definition used in recent publications has included the identification of recurrent GORD on pH studies instead of simply using subjective symptoms.^40–42^ However, this was not the case in most RCTs in our meta-analysis as many used patient-reported symptoms to define recurrence. This is justifiable because what matters clinically when considering treatment failure and the return of GORD is what matters symptomatically to the patient. Nevertheless, defining treatment failure in this way may have made more subtle differences between NF and TF harder to detect.

The surgical techniques also varied among the studies. For example, four of the included RCTs routinely placed a bougie in the oesophagus for calibration,^17,19,21,22^ before the wrap formation, whereas others did not.^16,23^ This can have a significant impact on the efficacy of the surgery and results should therefore be examined carefully.

According to this analysis, for most patients undergoing surgery for GORD, TF is recommended as the procedure of choice given that it appears to be as effective and durable as NF but with lower dysphagia rates. Patients with morbid obesity or existing oesophageal dysmotility should have a different setup and probably a more ‘tailored’ procedure.

## Conclusions

The available evidence regarding the optimal treatment of symptomatic GORD in patients without pre-existing oesophageal dysmotility disorder supports the long-term efficacy of partial posterior fundoplication (TF) as it provides equivalent outcomes in GORD symptom control with a lower risk of postoperative dysphagia compared with total fundoplication (NF). RCTs with a larger sample size and a follow-up duration of more than 15 years would be beneficial to justify the value of NF and TF on an even longer basis.
